# Geographic barriers to achieving universal health coverage: evidence from rural Madagascar

**DOI:** 10.1093/heapol/czab087

**Published:** 2021-07-31

**Authors:** Andres Garchitorena, Felana A Ihantamalala, Christophe Révillion, Laura F Cordier, Mauricianot Randriamihaja, Benedicte Razafinjato, Feno H Rafenoarivamalala, Karen E Finnegan, Jean Claude Andrianirinarison, Julio Rakotonirina, Vincent Herbreteau, Matthew H Bonds

**Affiliations:** MIVEGEC, University Montpellier, CNRS, IRD, 911 Avenue Agropolis, 34394 Montpellier, Montpellier, France; NGO PIVOT, BP23 Ranomafana, 312 Ifanadiana, Madagascar; NGO PIVOT, BP23 Ranomafana, 312 Ifanadiana, Madagascar; Université de La Réunion, UMR 228 Espace-Dev (IRD, UA, UG, UM, UR), 40 Av De Soweto, 97410 Saint-Pierre, Réunion, France; NGO PIVOT, BP23 Ranomafana, 312 Ifanadiana, Madagascar; NGO PIVOT, BP23 Ranomafana, 312 Ifanadiana, Madagascar; School of Management and Technological innovation, University of Fianarantsoa, BP 1135 Andrainjato, 301 Fianarantsoa, Madagascar; NGO PIVOT, BP23 Ranomafana, 312 Ifanadiana, Madagascar; NGO PIVOT, BP23 Ranomafana, 312 Ifanadiana, Madagascar; NGO PIVOT, BP23 Ranomafana, 312 Ifanadiana, Madagascar; Department of Global Health and Social Medicine, Harvard Medical School, 641 Huntington Avenue, Boston, Massachusetts 02115, USA; Ministry of Public Health, Ambohidahy, 101 Antananarivo, Madagascar; National Institut of Public Health, Befelatanana, 101 Antananarivo, Madagascar; Ministry of Public Health, Ambohidahy, 101 Antananarivo, Madagascar; Faculty of Medicine, BP. 375, 101 Antananarivo, Madagascar; Institut de Recherche pour le Développement, UMR 228 Espace-Dev (IRD, UA, UG, UM, UR), B.P. 86, Phnom Penh, Cambodia; NGO PIVOT, BP23 Ranomafana, 312 Ifanadiana, Madagascar; Department of Global Health and Social Medicine, Harvard Medical School, 641 Huntington Avenue, Boston, Massachusetts 02115, USA

**Keywords:** Community health, geographical information systems, healthcare utilization, health systems research, inequality

## Abstract

Poor geographic access can persist even when affordable and well-functioning health systems are in place, limiting efforts for universal health coverage (UHC). It is unclear how to balance support for health facilities and community health workers in UHC national strategies. The goal of this study was to evaluate how a health system strengthening (HSS) intervention aimed towards UHC affected the geographic access to primary care in a rural district of Madagascar. For this, we collected the fokontany of residence (lowest administrative unit) from nearly 300 000 outpatient consultations occurring in facilities of Ifanadiana district in 2014–2017 and in the subset of community sites supported by the HSS intervention. Distance from patients to facilities was accurately estimated following a full mapping of the district’s footpaths and residential areas. We modelled per capita utilization for each fokontany through interrupted time-series analyses with control groups, accounting for non-linear relationships with distance and travel time among other factors, and we predicted facility utilization across the district under a scenario with and without HSS. Finally, we compared geographic trends in primary care when combining utilization at health facilities and community sites. We find that facility-based interventions similar to those in UHC strategies achieved high utilization rates of 1–3 consultations per person year only among populations living in close proximity to facilities. We predict that scaling only facility-based HSS programmes would result in large gaps in access, with over 75% of the population unable to reach one consultation per person year. Community health delivery, available only for children under 5 years, provided major improvements in service utilization regardless of their distance from facilities, contributing to 90% of primary care consultations in remote populations. Our results reveal the geographic limits of current UHC strategies and highlight the need to invest on professionalized community health programmes with larger scopes of service.

**Key messages**

- Limited geographic access to primary care is one of the most important and hardest challenges for achieving UHC and improving population health in developing countries.
- There is little evidence on how health system change linked to UHC affects the geography of health access, leaving open questions about their effects on remote populations.
- We combined geographic data from hundreds of thousands of patient records from a rural district’s health system to identify the impact of a HSS intervention on geographic trends in access to care.
- Our results provide evidence of the substantial gaps in care that persist unless health systems integrate professional community health programmes with an expanded scope of services.

## Introduction

Despite considerable progress on the health-related development goals, every year five million children under 5 years die of treatable illnesses such as malaria, diarrhoea and respiratory infections. More than three and a half billion people lack access to essential health services (The World Bank, 2017; Fullman *et al.*, 2017). At the recent 40-year anniversary of the Alma Ata Declaration, 134 countries signed on to a renewed commitment to universal health coverage (UHC) based on a shared vision that primary health care and health services be ‘high quality, safe, comprehensive, integrated, accessible, available, and affordable for everyone everywhere’ (World Health Organization, 2018a). In practice, UHC policies tend to focus on financial coverage, such as through health insurance, in order to reduce out-of-pocket payments at health facilities, which are known to be barriers to care (Sachs, 2012; Garchitorena *et al.*, 2017; Dhillon *et al.*, 2011; Langlois *et al.*, 2016; Zombré *et al.*, 2017; Lagarde and Palmer, 2011; Johri *et al.*, 2014). However, there is growing recognition that among the greatest challenges to accessing health care are geographic barriers: terrain, waterways and other factors associated with physical distance between the patient and the service (Feikin *et al.*, 2009; McLaren *et al.*, 2014; Gething *et al.*, 2012; Noor *et al.*, 2003; Stock, 2012). The use of primary care decreases exponentially for populations living at increasing distance of primary healthcare centres (PHCs), known as the ‘distance decay’ effect (Feikin *et al.*, 2009; McLaren *et al.*, 2014; Gething *et al.*, 2012; Noor *et al.*, 2003; Stock, 2012). Distance decay in health access is equivalent to the effect of user fees (Bates *et al.*, 2012), which can be more directly reduced or eliminated (Sachs, 2012; Garchitorena *et al.*, 2017; Dhillon *et al.*, 2011; Langlois *et al.*, 2016; Zombré *et al.*, 2017; Lagarde and Palmer, 2011; Johri *et al.*, 2014).

While there is a considerable body of evidence on the relationship between health system access and user fees, there is surprisingly little evidence on the relationship between health system change and the geography of health access. Studies suggest that geographic barriers to PHC persist even when user fees have been removed, making these approaches insufficient to reach full population coverage of primary care services (Nguyen *et al.*, 2018; De Allegri *et al.*, 2011; Mills *et al.*, 2008). The leading policy strategy for addressing geographic barriers is through community health workers (CHWs); i.e. lay people who are trained to treat a subset of clinical cases (World Health Organization, 2018b; 2010). Yet, little is known about the effects of community health systems on the geography of health access or about their contribution towards the realization of universal access to primary health care. Can the leading policies designed to improve healthcare coverage—UHC and community health—actually overcome these key barriers?

In most developing countries, national policies consider CHWs as local volunteers, with minimum requirements of formal education. Compensation for CHWs is well below the national minimum wage and is frequently based on a social marketing strategy, where CHWs earn a markup for the sale of medicines. Community-based diagnosis and treatment is generally restricted to malaria, pneumonia and diarrhoea for children under five (Ahmed *et al.*, 2010). The burden of disease thus remains unmet for the large majority of the population, even when community health systems are fully functioning. New World Health Organization (WHO) guidelines, not yet fully adopted by most countries, recommends paying CHWs minimum wage, removing use fees and providing dedicated supervision (World Health Organization, 2018b), but there remains debate on how to optimize community health.

The situation of Madagascar is illustrative of the challenges of many low-income countries attempting to translate international policies for UHC and community health to the national level in a context of limited resources. This island nation is one of the poorest countries in the world, with among the least well-funded health systems (World Bank, 2019). In 2014, Madagascar had less than three doctors, nurses and midwives per 10 000 people (World Health Organization, 2019), with a lower concentration in rural areas, where over three-quarters of the population live (Institut National de la Statistique, 2009). Access to health care is particularly low for the majority of the population living more than 5 km away from a PHC (Garchitorena *et al.*, 2017; World Health Organization, 2019; Kashima *et al.*, 2012). To address this, the country has significantly increased health spending in recent years and, in 2015, it signed a national policy for UHC that is currently in its pilot phase (Government of Madagascar, 2015). Yet, the contribution of CHWs to improving primary care access in Madagascar is limited, since CHWs work on a voluntary basis, manage mostly illnesses of early childhood, and significant challenges remain to support their activities (e.g. supervision and procurement).

Here, we take advantage of a natural experiment in global health, where an integrated, district-level health system strengthening (HSS) intervention aimed at achieving universal coverage at the local level was implemented in a rural district of Madagascar, ahead of the national scale up of the UHC strategy. Starting in 2014, a non-governmental organization (NGO), partnered with the Government of Madagascar to establish a model health system in the southeastern district of Ifanadiana (∼200 000 people). A range of HSS programmes were initiated in a third of the district (see Supplementary Table S1), removing user fees at health facilities, ensuring health system readiness (infrastructure, personnel and supply chain), improving clinical programmes (maternal and child health) and supporting integrated information systems at all levels of care (community health, primary care facilities and the district hospital). Early results showed improved quality of primary care (Ezran *et al.*, 2019), a tripling of facility utilization rates (Garchitorena *et al.*, 2018), and declines in under-five and infant mortality rates in the first 2 years of intervention (Garchitorena *et al.*, 2018). Later, in 2016, these programmes were extended to include strengthened CHWs—who were trained, supervised and equipped (Cordier *et al.*, 2020; Bonds *et al.*, 2017).

With a unique geographically explicit patient-level data set encompassing all health centre visits in the district during 4 years, the aim of this study was to examine the effect of increasing financial coverage and strengthening the public health system on the geographic access (community *vs* facility-based) to primary health care. The ultimate goal was to provide evidence, via this district-level pilot, on the contribution and limitations of broader policies for UHC and community health towards the realization of universal access to primary health care in rural settings of developing countries.

## Methods

### Study site

Ifanadiana is a rural health district of approximately 200 000 people located in the region of Vatovavy-Fitovinany, in Southeastern Madagascar. As per Ministry of Health (MoH) norms, Ifanadiana district has one reference hospital, one main primary care health centre (PHC2) for each of its 13 communes (subdivision of a district with ∼15 000 people), six additional basic health centres for its larger communes (PHC1), and one community health site with two CHWs for each of its 195 fokontany (subdivision of a commune with ∼1000 population). The integrated HSS intervention carried out by the MoH–NGO partnership (summarized in Supplementary Table S1) began in 2014, is guided by existing MoH policies, covers all six WHO building blocks of HSS and is implemented across all three levels of care in the district (community, health centre and hospital). This intervention is structured through the integration of horizontal improvements in system ‘readiness’, vertically aligned clinical programmes and information systems. Readiness includes infrastructure and sanitation, staffing and equipment to improve the quality of care; procurement systems; an ambulance network; the removal of user fees and provision of social support to patients; trainings and frequent supervision of health staff. The clinical programmes include malnutrition and integrated management of child illness through strengthened community health programmes, PHCs and hospital (details can be found in Garchitorena *et al.*, 2018; Bonds *et al.*, 2017). The core activities in the first years (2014–2017) covered approximately one-third of the population of Ifanadiana (referred to as ‘HSS catchment’), with some activities such as medical staff recruitments spanning the whole district (Supplementary Table S1).

In addition to the HSS intervention, the population of Ifanadiana benefited from two other notable programmes that covered both the HSS catchment and the rest of the district (RoD) in this period. The PAUSENS project, funded by the World Bank and implemented in 2013–2017, provided a basic package of services free of charge in all 13 PHC2 for every woman attending the health centre for antenatal, delivery or postnatal care (first 6 weeks) and children under age five with any illness (The World Bank, 2012). The project also included training, support for child vaccination in remote areas and some equipment to health centres. The Mikolo project, funded by U.S. Agency for International Development and implemented in 2012–2017, provided support to a network of 150 CHWs in the remote fokontany (further than 5 km from a health centre) of eight communes in Ifanadiana, four of which were in the HSS catchment and four in RoD. The project organized annual trainings and periodic supervision and provided some equipment, supplies and an initial stock of medicines to each CHWs. The main difference between HSS catchment and RoD (our control group) was the implementation of the HSS intervention by the MoH–NGO partnership.

### Health system utilization data

From January 2014 to December 2017, we obtained data from the registries on all individuals attending any PHC for an outpatient consultation in the district. The data were collected via regular visits to each PHC in the district by the NGO staff every 3–4 months, in agreement with the head of each PHC and the district medical inspector. This allowed for the creation of a patient-level, de-identified digital database. For each patient (new visits; follow-up excluded), information included the age, name of the fokontany of residence and malaria status. Fokontany are the smallest administrative units, composed of one or several villages, and are located at varying distances from the nearest PHC (0–20 km). For the period from January to December 2017, we also collected consultation data from the community health sites supported by the MoH–NGO partnership at this time (four out of five communes in the intervention area and 43 fokontany with an estimated population of about 55 000). This information, which was already available at the fokontany level, was obtained from the monthly report to the district and was verified for data quality and corrected where necessary by the NGO’s monitoring and evaluation team.

Population data for each fokontany was obtained from the MoH. Consistent with MoH estimates, the population of children under 5 years was set at 18% of the total catchment population. Although official population data are sometimes deemed inaccurate, we previously showed that estimating catchment populations using available data for our district from other recognized sources such as WorldPop (2017) did not change the results of per capita utilization rates analyses (Garchitorena *et al.*, 2017). Information about key dates of the HSS intervention, especially the beginning of the user-fee exemption programme and the community health programme for each supported commune, were obtained from internal records within the NGO. Number of health professionals at each PHC per month were obtained from district’s records and Service Availability and Readiness Assessments (Ezran *et al.*, 2019).

### Geographic information system

We gathered geographic information from multiple sources in order to estimate the distance and travel time from each house in Ifanadiana district to the nearest PHC. First, we mapped all footpaths, residential areas, houses and rice fields in the district using very-high-resolution satellite images available through OpenStreetMap (OSM). For this, we implemented a participatory approach in collaboration with the non-profit organization Humanitarian OpenStreetMap Team (HOT). The district was divided in tiles of 1 km by 1 km and a request for mapping them was made publicly available through the HOT website (Humanitarian OpenStreetMap Team, 2019). Mapping was carried out in a two-stage process, where tiles that had been mapped had to be validated by a separate contributor. Most tiles were mapped and validated by a dedicated team hired through the project to ensure data quality and completion within the project deadlines. When mapping was completed, we used the Open Source Routing Machine (OSRM) engine to query our OSM data and accurately estimate the shortest path between each house in the district and the nearest PHC.

Second, to estimate travel speed by foot under different terrain and environmental conditions, we conducted field global positioning system (GPS) tracking between September 2018 and April 2019 in a sample of itineraries in Ifanadiana. A total of 168 itineraries by foot amounting to nearly 1000 km were collected by the NGO’s community and research teams, in collaboration with CHWs. For this, we used the android mobile app ‘OSMAnd’ installed in tablets (Samsung Galaxy A10.1) and we recorded every 10 s the GPS coordinates, time and altitude.

Third, we built remotely sensed land cover maps combining information from Sentinel-2 satellites and OSM. We integrated land cover maps with the rest of graphical information system (GIS) data (climate, elevation, etc.) to statistically model travel speed and estimate terrain characteristics associated with higher or lower speed using a generalized additive mixed model. We finally combined model results, GIS data and the shortest paths estimated by OSRM in order to predict travel time to seek care at the nearest PHC for every house in Ifanadiana. The aggregated distance and travel time for a fokontany was the average of all houses in the fokontany. A detailed description of the methods used to estimate distance and travel time to PHC is available in Ihantamalala *et al.* (2020).

### Data analysis of health system data

**Figure 1.**
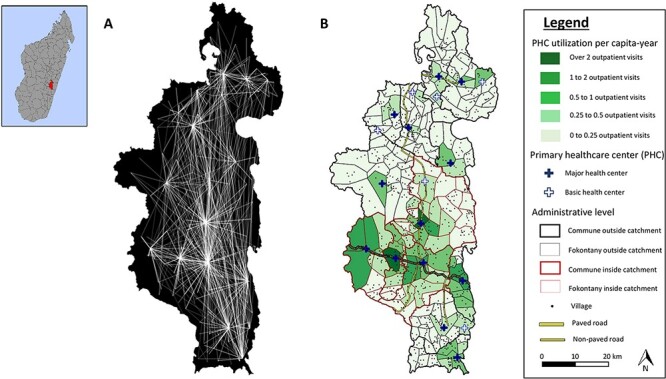
Geographic origin of patients from the 19 PCH in Ifanadiana district, 2014–2017. (A) Origin of all patients attending each of the 19 health centers in Ifanadiana district. For improved visualization, lines between Fokontany and PHC are included only when they represent over 100 patients, with line transparency inversely proportional to the logarithm of the number of visits. These data were aggregated to obtain a total number of per-capita visits per month for each Fokontany (lowest administrative unit, comprising one or several villages). (B) Average number of PHC visits per capita-year for each Fokontany during the study period.

The impact of the HSS intervention on utilization rates at each fokontany was modelled using interrupted time-series analyses with control groups (Kontopantelis *et al.*, 2015). For this, we first aggregated health centre patient-level information to estimate per capita utilization rates per month for each Fokontany in Ifanadiana district (Figure 1). We studied the linear and non-linear effect of travel distance and travel time from each fokontany to the nearest PHC on utilization rates. We assessed the impact of two programmes designed to reduce financial barriers (i.e. user-fee exemption) and to reduce geographic barriers (i.e. community programme) by assessing the level of change in utilization (immediate impact) associated with each programme (Kontopantelis *et al.*, 2015). We hypothesized that the community programme could have a positive impact on facility-based PHC utilization via increased sensitization, awareness and referrals by CHWs, especially for the 82% of the population over 5 years of age that is beyond the scope of CHWs work. We also controlled for linear and seasonal trends in utilization rates in the absence of the programmes; for baseline differences in HSS-supported PHC and in the type of PHC (PHC1 or PCH2); and for the number of health staff over time in the closest PHC for each fokontany.

Per capita utilization rates at PHC were modelled for each fokontany using binomial regressions in generalized linear mixed models, with a random intercept introduced for the closest PHC. All other variables were introduced as fixed effects. Each explanatory variable was studied through univariate analyses and those with *P*-values below 0.1 were included in multivariate models. Orthogonal polynomial terms of degree 2 were included to account for the non-linear relationship of per capita utilization with travel distance/time to the PHC. We included interaction terms between the HSS programmes and the travel distance/time to the PHC in the multivariate model to test whether these programmes had a different effect on remote populations. Model selection was performed through step-wise procedures based on Akaike information criterion (AIC), by selecting the reduced model with the lowest AIC. Model assumptions in the final model were verified, including violations to homogeneity and independence of residuals. We introduced a 1-month utilization lag in the final models to remove temporal autocorrelation in the residuals. To facilitate interpretation of results, we report exponentiated model coefficients, which reflect the ratio of change (odds ratio, OR) in utilization rates associated with each explanatory variable. Several sets of analyses were carried out in order to study PHC utilization separately in the general population, in specific age groups (children under five), and including or excluding malaria cases from the analyses. Using the final model for the general population, we predicted PHC utilization for every Fokontany in Ifanadiana under a scenario without HSS programmes (no user-fee exemption, no community health support and three health staff per PHC) and with HSS programmes (user-fee exemption, community health support and seven health staff per PHC). Finally, we compared geographic trends in primary care when combining utilization at both PHC and community health sites in the subset of 43 fokontany where the community health programme had been strengthened by the MoH–NGO partnership. Analyses were performed with R software (R Development Core Team, 2011) and R packages ‘lme4’, ‘gstat’, ‘rgdal’ and ‘ggplot2’.

## Results

### PHC utilization by geographic proximity

Of the 314 443 patients who attended a PHC for an outpatient visit, 276 865 patients had a known geographic location and 99.25% of these (274 798) came from within the district (Supplementary Figure S1). Table 1 presents summary statistics of the patient population based on these geographic analyses. Although more than two-thirds of the population lived further than 5 km from a PHC (5–22 km) and 27% lived further than 10 km (10–22 km), these populations represented only 40% and 9% of all patient visits, respectively. Only a fourth of the population lived within 1 h of a PHC. Average annual PHC utilization per capita rates were nearly triple inside the HSS intervention catchment (0.64) than in the RoD (0.23) for all ages and more than double for children under 5 years. Utilization rates more than halved for every 5 km and every hour of travel from a PHC for every age group considered (Table 1).

**Table 1.** Geographic distribution of populations and PHC outpatient visits in Ifanadiana district, 2014–2017

		HSS Catchment		Distance to PHC			Time to PHC		
	District	Inside	RoD	0–5 km	5–10 km	10–22 km	0–1 h	1–2 h	2–5 h
Population (prop.)									
All ages	198 175	72 152 (0.36)	126 023 (0.64)	63 811 (0.32)	80 790 (0.41)	53 573 (0.27)	49 269 (0.25)	74 169 (0.37)	74 738 (0.38)
Under 5 years old	35 671	12 987 (0.36)	22 684 (0.64)	11 486 (0.32)	14 542 (0.41)	9643 (0.27)	8868 (0.25)	13 350 (0.37)	13 453 (0.38)
Over 5 years old	162 503	59 164 (0.36)	103 338 (0.64)	52 325 (0.32)	66 248 (0.41)	43 930 (0.27)	40 400 (0.25)	60 818 (0.37)	61 285 (0.38)
Total number of patients (prop.)									
All ages	270 747	173 497 (0.64)	97 250 (0.36)	163 656 (0.6)	83 667 (0.31)	23 424 (0.09)	145 249 (0.54)	83 154 (0.31)	42 344 (0.15)
Under 5 years old	92 533	52 240 (0.56)	40 293 (0.44)	52 761 (0.57)	31 308 (0.34)	8464 (0.09)	45 576 (0.49)	31 356 (0.34)	15 601 (0.17)
Over 5 years old	178 214	121 257 (0.68)	56 957 (0.32)	110 895 (0.62)	52 359 (0.29)	14 960 (0.08)	99 673 (0.56)	51 798 (0.29)	26 743 (0.15)
Per capita utilization per year									
All ages	0.39	0.64	0.23	0.74	0.29	0.12	0.84	0.32	0.16
Under 5 years old	0.74	1.07	0.53	1.33	0.6	0.25	1.46	0.67	0.33
Over 5 years old	0.31	0.54	0.16	0.61	0.22	0.1	0.70	0.24	0.12

**Figure 2.**
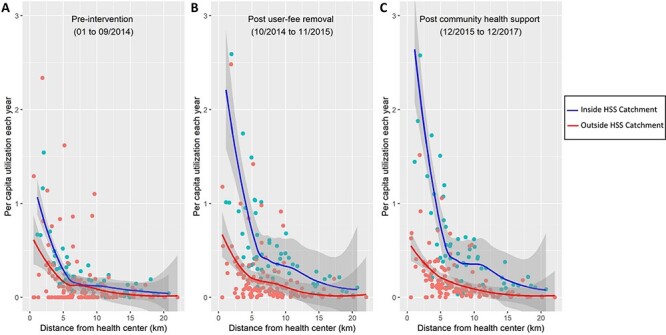
Average PHC per capita utilization by distance of Fokontany to PHC. Colors represent the HSS intervention catchment (blue) and the rest of Ifanadiana district (blue). Each dot represents one of the 195 Fokontany in Ifanadiana, solid lines are the respective non-linear smooth (local regression, LOESS method) and grey shades are the 95% confidence intervals around each smooth. A clear distance decay pattern can be observed, accentuated for the HSS intervention catchment due to a larger increase in utilization near PHC following the HSS intervention (B and C). To improve visualization, one dot from the HSS intervention catchment post intervention (4.5 per capita-year) was removed.

Spatial analyses revealed that utilization rates increased over time in the HSS intervention catchment but declined dramatically as distance increased within the first 5 km from a PHC, especially after the system was strengthened at the facility level (Figure 2). The HSS intervention exacerbated the impact of geography on utilization (Figure 2b and c), but the ratio in PHC utilization between populations living in close proximity (<2.5 km) *vs* those leaving the furthest (>10 km) remained the same at over 10 times higher. Following user-fee exemptions, the HSS intervention catchment experienced a substantial increase in utilization, from 1 to nearly 3 visits per capita year for populations living in close proximity to a PHC and from 0.25 to about 0.5 visits per capita year for populations living 5–10 km from a PHC (Figure 2b and c).

**Figure 3.**
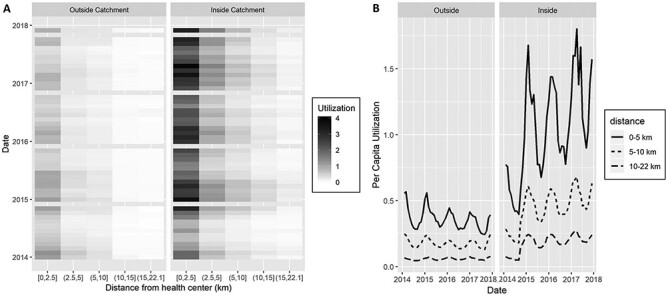
Time-series of PHC per capita utilization by distance of Fokontany to PHC, inside and outside the HSS intervention catchment. (A) Heat map of observed monthly PHC utilization, where grey color scale is proportional to average per capita values at each distance. (B) Model predictions of PHC per capita utilization for Ifanadiana, aggregated by intervention catchment area and distance to PHC. Both graphs show that implementation of HSS activities in the intervention catchment resulted in a shift by 5km of PHC utilization patterns (e.g. those living 5-10km from a PHC in the intervention catchment, have similar utilization rates than those within 5km outside the catchment). Utilization rates were annualized to improve comparability of results.

Despite strong seasonal variation in utilization rates, particularly for populations living close to a PHC, trends remained unchanged in the RoD during the study period (Figure 3). Compared with the RoD, HSS activities in the intervention catchment resulted in a shift by 5 km in PHC utilization patterns so that populations living 5 km further from a strengthened PHC accessed care at rates comparable to those living 5 km closer to a facility that did not receive the intervention (Figure 3). We also observed seasonality in the average distance patients travelled to access a PHC during the year (Supplementary Figure S2). Overall, 50% of outpatient visits seen in the intervention catchment came from localities within 2 km of a PHC and over 75% from localities within 4 km, but in the months of May through July (dry season) patients came from further away. These seasonal patterns were not observed in the RoD (Supplementary Figure S2).

### Impact of HSS programmes on PHC utilization

Our models confirmed the exponential decrease in PHC utilization due to geographic distance after accounting for programme implementation, health system factors and underlying temporal trends (Table 2, Supplementary Figure S3). We carried out several models to understand the consistency of associations when including or excluding malaria cases (because of their influence on PHC utilization seasonality), as well as for populations of all ages or only children under 5 years. In every model, a non-linear relationship with distance to PHC best explained utilization patterns (better than using a linear relationship with distance or using travel time as the explanatory variable), and this was the most important variable associated with PHC utilization trends (Supplementary Table S2). After controlling for time trends and baseline differences in health system factors, patterns of geographic utilization of healthcare services were also highly sensitive to HSS programmes implemented in this period, especially the fee-exemption programme to increase financial access to PHC and the community program to address geographic barriers (Table 2). Both programmes had a positive impact on PHC utilization rates for all ages (OR = 1.09 and OR = 1.14, respectively), with a higher relative increase for those populations living further away (OR = 1.45 and OR = 1.09, respectively, every 10 km from a PHC). These results were consistent regardless of the age group considered or whether malaria cases were included in the model (Table 2). Our models accurately explained spatial and temporal utilization patterns at PHC (Supplementary Figure S4), allowing us to predict dynamics of PHC geographic utilization in the district (S2 Video).

**Table 2.** Multivariate model results (generalized linear mixed models with random intercept at the PHC closest to the fokontany of residence)

	Outpatient visits for all ages	Outpatient visits for children under age 5	Outpatient visits for all ages, excluding malaria	Outpatient visits for children under age 5, excluding malaria
Variable	OR (95% CI)	OR (95% CI)	OR (95% CI)	OR (95% CI)
Intercept (visits per capita month)	0.02 (0.011–0.037)	0.032 (0.019–0.054)	0.015 (0.008–0.03)	0.024 (0.013–0.043)
Geographic factors				
Network distance to PHC (10 km, linear)^a^	0.126 (0.121–0.131)	0.228 (0.212–0.244)	0.095 (0.091–0.1)	0.141 (0.13–0.154)
Network distance to PHC (10 km, quadratic)^a^	1.102 (1.076–1.13)	0.898 (0.862–0.937)	1.261 (1.226–1.297)	1.07 (1.018–1.126)
Health system factors				
Number of health staff	1.042 (1.037–1.048)	1.032 (1.023–1.041)	1.043 (1.037–1.049)	1.039 (1.029–1.048)
Major PHC (*vs* basic PHC)	3.238 (1.5–6.993)	3.449 (1.764–6.744)	3.16 (1.421–7.024)	3.634 (1.762–7.493)
HSS catchment (*vs* outside)	0.663 (0.636–0.69)	0.637 (0.597–0.68)	0.641 (0.611–0.672)	0.634 (0.587–0.684)
Impact of HSS programmes				
User-fee exemption programme	1.09 (1.063–1.118)	0.946 (0.908–0.987)	1.18 (1.145–1.215)	–
Interaction with distance to PHC (10 km)^a^	1.454 (1.433–1.476)	1.416 (1.385–1.448)	1.44 (1.415–1.466)	1.361 (1.328–1.394)
Community health programme	1.147 (1.125–1.17)	1.148 (1.118–1.179)	1.112 (1.087–1.137)	1.081 (1.039–1.125)
Interaction with distance to PHC (10 km)^a^	1.095 (1.08–1.111)	–	1.127 (1.109–1.145)	1.052 (1.02–1.085)
Underlying trends				
Linear trend (year)	0.968 (0.962–0.973)	0.968 (0.959–0.977)	0.988 (0.981–0.995)	–
Seasonal trend^b^	1.204 (1.197–1.211)	1.212 (1.199–1.225)	1.031 (1.024–1.038)	1.127 (1.114–1.141)
Lagged trend (1-month lag)^c^	1.473 (1.466–1.48)	1.304 (1.297–1.311)	1.65 (1.637–1.663)	1.363 (1.352–1.375)

a The variable network distance represents tens of kilometres (distance in km × 10^−1^) to facilitate interpretation of coefficients and enable model convergence.

b Seasonal trend was constructed as [sin(2π(Month_i_ + θ/12], where θ was the horizontal shift that best fit the data of each model.

c Lagged trend transformed into visits per capita year to allow interpretation of results.

**Figure 4.**
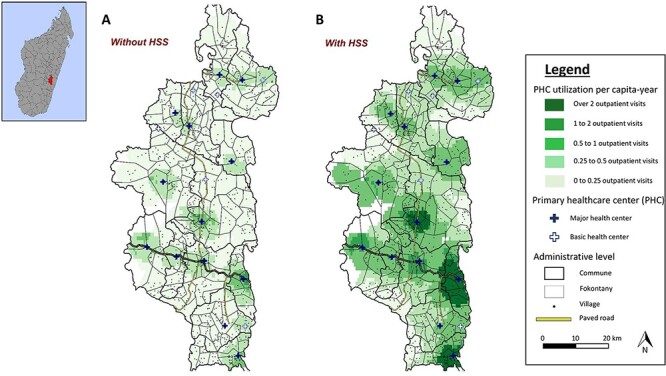
Predictions of geographic distribution of PHC per capita utilization in Ifanadiana district according to scenarios of HSS intervention implementation. Color shades represent predictions of annual PHC per capita visits in (A) a scenario where no HSS activities are implemented, and (B) a scenario where HSS are implemented in the whole district. Maps reveal that despite improvements, even if HSS were implemented across the district, a large proportion of the population would remain with very low levels of realized access to facility-based primary care.

Predictions from the model for all ages suggested that in the absence of these programmes, only 1% of the population in Ifanadiana district would have per capita PHC utilization of one visit or more per year and 12% would have 0.5 visits or more per year. If these programmes were implemented everywhere in the district, nearly one-quarter of the population (23%) would have a PHC utilization of at least one visit and nearly half (47%) would have at least 0.5 visits per capita year. Maps in Figure 4 show predictions of the geographic distribution of PHC utilization with and without implementation of HSS programmes, revealing substantial gaps in health system coverage for remote populations. PHC utilization remained low for remote populations under a variety of HSS scenarios that included hiring additional health staff at PHC, removing user fees and strengthening community health (Supplementary Figure S5).

### Utilization for children under 5 years when combining PHC and community health consultations

**Figure 5.**
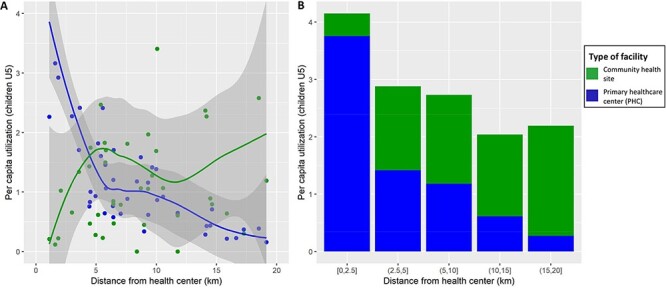
Annual utilization rates of primary care (PHC and community health sites) per capita by children under 5 years in the HSS intervention catchment, 2017. Graphs show per capita utilization at different distances to the nearest PHC, disaggregated by PHC and community health visits. It reveals that utilization at community health sites compensates the distance decay observed for PHC use, with higher community health site utilization at further distance to PHC and reaching over 2 visits per capita-year for all distance groups. To improve visualization, four dots were removed in the left graph (utilization over 4 per capita-year).

To reduce geographic barriers to care, CHWs (two per fokontany) can manage childhood illnesses such as malaria, diarrhoea or pneumonia for children under 5 years of age. Data from community health sites in four communes of the HSS intervention area revealed that when combining outpatient visits from both PHC and community health sites for children under five, utilization of primary care in this period exceeded one visit per child for 39 of the 43 fokontany (94% of under-five population), regardless of the distance of the population to a PHC (Figure 5a). On average, combined utilization exceeded two visits per child per year in all distance groups from a PHC (Figure 5b). Average utilization at community health sites substantially increased at further distances from a PHC: annual utilization was less than 0.5 at 2.5 km from a PHC and nearly two at fokontany more than 15 km from a PHC. As a result, visits at community health sites accounted for 90% of total primary care visits in fokontany further than 15 km from a PHC, while they accounted for only 10% of total visits at 2.5 km or less from a PHC (Figure 5b). Combined utilization of primary care was still lower for children living further away from a PHC, but the effect of distance was much less pronounced due to the exponential increase in community health site utilization at higher distances from PHC (Figure 5a), which essentially compensated for the distance decay.

## Discussion

A renewed commitment to strengthening primary healthcare systems and ensuring UHC is essential to meet the health-related Sustainable Development Goals (United Nations General Assembly, 2015), but enormous questions remain on how to do this (World Health Organization, 2018b; Stenberg *et al.*, 2017; Giedion *et al.*, 2013; Atun *et al.*, 2008; Hatt *et al.*, 2015). Here, we analysed geographic data from hundreds of thousands of patients in a rural district of Madagascar undergoing health system transformation to understand how facility- and community-based interventions contribute to health system coverage. Our results reveal that facility-based primary care has limited geographic coverage, even when it is free of charge at the point of service and of improved quality, the focus of most national UHC policies (Garchitorena *et al.*, 2017; Ezran *et al.*, 2019). Communities that lived within 5 km of a supported PHC exceeded one visit per person year, but the intervention accentuated the distance decay (exponential decrease) in PHC utilization and widened the gap with remote populations, which exacerbated disparities. We predict that scaling up PHC interventions alone (removing user fees and improving health system readiness) would only achieve modest increases in geographic coverage, with three-fourths of the population consulting at facilities less than once per person year. Strengthening community health can have substantial impacts on the geographic reach of the health system. The effect of geography on primary care access was greatly reduced for children under 5 years when considering community health consultations, reaching over two consultations per child year regardless of distance. CHWs were the main source of healthcare delivery for children in remote populations, representing 90% of primary care visits for those living further than 15 km from a PHC.

Research on geographic accessibility to care has generally focused on characterizing either potential access (population within a certain distance from a PHC) or realized access (actual utilization at different distances to a PHC) (Yao and Agadjanian, 2018; Chukwusa *et al.*, 2019). In terms of potential access, a travel time of 1 or 2 hours to health services is a typically accepted measure of accessibility to health services (Gething *et al.*, 2012; Pilcher *et al.*, 2014; Noor *et al.*, 2006; Juran *et al.*, 2018; Bailey *et al.*, 2011). We estimated distance to PHC using a complete district mapping of over 20 000 km of footpaths and 100 000 houses. We then parametrized travel time with hundreds of hours of fieldwork and remote sensing analyses. This approach allowed us to improve on previous methods in developing countries that use either Euclidean distances, friction surfaces (Stock, 2012; Makanga *et al.*, 2016; Munyaneza *et al.*, 2018) or self-reported answers in surveys (Gething *et al.*, 2012; Noor *et al.*, 2003; Al-Taiar *et al.*, 2010). We found that the majority of the population in Ifanadiana district (75%) lived more than 1 h from primary care at a PHC and over one-third (38%) lived further than 2 h. These figures are significantly worse than regional estimates of primary healthcare access in sub-Saharan Africa (Weiss *et al.*, 2020) and more comparable to estimated access to secondary care at hospitals in the region (Juran *et al.*, 2018; Ouma *et al.*, 2018). This may suggest that either access to care in Ifanadiana is indeed far worse than average or multi-country approaches tend to underestimate the proportion of the population with poor access to care, or both.

When user fees were removed and HSS activities were in place, we found that utilization rates reached between one and three consultations per person year for populations in close proximity to PHC, similar to findings in other studies in Africa (Zombré *et al.*, 2017), and close to utilization rates in many OCDE countries with lower disease burdens (Consultations with doctors, 2018). This suggests that UHC policies can be effective at increasing healthcare use to internationally acceptable levels for some populations. Yet, although distance to PHC is not always associated with lower utilization or worse outcomes (Munyaneza *et al.*, 2018; Lankowski *et al.*, 2014; Gething *et al.*, 2004), we observed a similar distance decay in utilization as previously described in other settings (Feikin *et al.*, 2009; McLaren *et al.*, 2014; Gething *et al.*, 2012; Noor *et al.*, 2003; Stock, 2012; Kelly *et al.*, 2016). Moreover, we show that this decay can be even more pronounced once interventions aimed at increasing healthcare access have been implemented. We found that the median distance of patients to PHC following the HSS was 2 km, similar to results found in a rural area of Western Kenya (Feikin *et al.*, 2009). Our results are also consistent with evidence from Burkina Faso and Ghana, where user-fee exemptions and HSS strategies achieved greater equity across socio-economic groups but did not overcome geographic barriers (Langlois *et al.*, 2016; De Allegri *et al.*, 2011; Mills *et al.*, 2008; De Allegri *et al.*, 2015; Hounton *et al.*, 2008). This puts into question the assumption that UHC policies alone, when they are in place and effective, can ensure the provision of primary healthcare services ‘for everyone, everywhere’.

Using geographic information from nearly 300 000 primary care visits to PHC, we show that health system data can allow for powerful studies of spatio-temporal changes in healthcare access and for drawing key insights to improve UHC strategies. Previous studies that combined measures of geographic access with healthcare utilization or service coverage have been restricted to discrete services or conditions such as obstetric care, tuberculosis, malaria and HIV (Noor *et al.*, 2003; 2006; Munyaneza *et al.*, 2018; Lankowski *et al.*, 2014; Ebener *et al.*, 2015; Kuupiel *et al.*, 2019). Electronic health management information system (HMIS) data currently available rarely include a low level of geographic disaggregation, so studies typically use information from national surveys or restrict the extraction of HMIS geographic information to particular conditions (Langlois *et al.*, 2016; McLaren *et al.*, 2014; De Allegri *et al.*, 2011; 2015; Buor, 2003; Rosero-Bixby, 2004; Ruktanonchai *et al.*, 2016) or to small samples of patients (Stock, 2012; Gething *et al.*, 2004). One of the most precise studies linked over 3000 paediatric health visits in seven clinics in Kenya to individual identifiers from a demographic surveillance system (Feikin *et al.*, 2009). However, a push for electronic data collection to improve health information systems is underway in many developing countries, thanks to the scale up of the open source DHIS2 (District Health Information Software) among other platforms, which can be combined with community-based mobile tools for registering cases and track patient-level data at different levels of care (Dehnavieh *et al.*, 2018). The level of granularity and timeliness of data of these e-health platforms will open new possibilities for integration of feedback loops between spatial modelling approaches in local planning and implementation of health strategies to maximize geographic access.

Our study had several limitations. First, our estimates of travel time were based on speeds recorded in fieldwork done by health workers and community members, so they represent local travel time for healthy individuals. Other groups such as ill individuals, pregnant women or the elderly likely take longer to reach health facilities, and factors such as break time during a route that were not considered here could be particularly relevant for long distances. As a result, even our locally calibrated results on travel time may be underestimating true travel time for certain vulnerable groups. Second, in this study we assessed aggregate changes in per capita utilization by fokontany and compared differences between catchment areas rather than evaluating individual patient itineraries. A study on prenatal care in Mozambique showed that although most women living near a PHF used the closest facilities, those who lived more than 5.5 km away could travel to a further PHF to seek better services (Yao and Agadjanian, 2018). If patients from outside the HSS catchment preferred to attend a HSS-supported PHC despite further distance, this could have resulted in an underestimation of the intervention effects. Third, studies in Ghana and Nigeria found that the distance decay was more important for illiterate, low-income, women, children and elderly populations (Stock, 2012; Buor, 2003), but we could only test for the decay in different age groups because it was the only demographic information collected. Finally, like for any local study, the generalizability of results presented here may be limited to other rural, low-income settings with similar characteristics as Ifanadiana in terms of geography, socio-economic level and health system factors. Further research is needed to assess whether similar effects of UHC and community health policies on primary care access are observed elsewhere.

In conclusion, the results from this study have important implications for the UHC strategy in Madagascar and other low-income countries, suggesting that wider support to community health may be necessary to achieve universal access to primary care. Although there remains debate on how to optimize community health, a greater ability for populations to reach facilities is critical in order to directly address the geographic burden of disease, and professionalized CHWs could contribute to this by expanding the scope of primary care services they provide across a greater range of clinical cases and demographic groups. More generally, we show how a model system in global health—based on the dynamic integration of data and services at multiple levels of the health system in geographically constrained areas—can help address fundamental questions on key global health policy issues.

## Supplementary Material

czab087_Supp

## Data Availability

Data are available upon request to the address research@pivotworks.org.

## References

[R1] Ahmed HM , Mitchell M, Hedt B. 2010. National implementation of Integrated Management of Childhood Illness (IMCI): policy constraints and strategies. *Health Policy* 96: 128–33. 20176407 10.1016/j.healthpol.2010.01.013

[R2] Al-Taiar A , Clark A, Longenecker JC, Whitty CJM. 2010. Physical accessibility and utilization of health services in Yemen. *International Journal of**Health**Geographics* 9: 1–8. 10.1186/1476-072X-9-38 PMC2914054 20663146

[R3] Atun RA , Bennett S, Duran A. 2008. When do vertical (stand-alone) programmes have a place in health systems? *WHO European Ministerial Conference on Health Systems*: 1–28. https://apps.who.int/iris/handle/10665/107977.

[R4] Bailey PE , Keyes EB, Parker C et al. 2011. Using a GIS to model interventions to strengthen the emergency referral system for maternal and newborn health in Ethiopia. *International Journal of Gynecology & Obstetrics* 115: 300–9. 21982854 10.1016/j.ijgo.2011.09.004

[R5] Bates MA , Glennerster R, Gumede K, Duflo E The price is wrong. *Field Actions Science Reports* 2012. Special issue 4. http://factsreports.revues.org/1554.

[R6] Bonds MH , Garchitorena A, Cordier L et al. 2017. Advancing a science of sustaining health: a new platform for a model district in Madagascar. *bioRxiv*: 141549. doi: 10.1101/141549.

[R7] Buor D . 2003. Analysing the primacy of distance in the utilization of health services in the Ahafo-Ano South district, Ghana. *The International Journal of**Health**Planning and Management* 18: 293–311. 10.1002/hpm.729 14727709

[R8] Chukwusa E , Verne J, Polato G et al. 2019. Urban and rural differences in geographical accessibility to inpatient palliative and end-of-life (PEoLC) facilities and place of death: a national population-based study in England, UK. *International Journal of**Health**Geographics* 18: 8. 10.1186/s12942-019-0172-1 PMC6503436 31060555

[R9] Consultations with doctors . 2018. *Health at a Glance 2017: OECD Indicators*. Paris: OECD Publishing, 168–9. doi: 10.1787/health_glance-2017-en.

[R10] Cordier LF , Kalaris K, Rakotonanahary RJL et al. 2020. Networks of care in rural Madagascar for achieving universal health coverage in Ifanadiana district. *Health Systems & Reform* 6: 2–e1841437. doi: 10.1080/23288604.2020.1841437. 33314984

[R11] De Allegri M , Ridde V, Louis VR et al. 2011. Determinants of utilisation of maternal care services after the reduction of user fees: a case study from rural Burkina Faso. *Health Policy* 99: 210–8. 21056505 10.1016/j.healthpol.2010.10.010

[R12] De Allegri M , Tiendrebéogo J, Müller O et al. 2015. Understanding home delivery in a context of user fee reduction: a cross-sectional mixed methods study in rural Burkina Faso. *BMC Pregnancy Childbirth* 15: 330. 10.1186/s12884-015-0764-0 PMC4676832 26653013

[R13] Dehnavieh R , Haghdoost AA, Khosravi A et al. 2019. The District Health Information System (DHIS2): a literature review and meta-synthesis of its strengths and operational challenges based on the experiences of 11 countries. *Health Information Management Journal* 48: 62–75. doi: 10.1177/1833358318777713. 29898604

[R14] Dhillon RS , Bonds MH, Fraden M, Ndahiro D, Ruxin J. 2011. The impact of reducing financial barriers on utilisation of a primary health care facility in Rwanda. *Global Public Health* 2007: 1–16. 10.1080/17441692.2011.593536 PMC3227794 21732708

[R15] Ebener S , Guerra-Arias M, Campbell J et al. 2015. The geography of maternal and newborn health: the state of the art. *International Journal of**Health**Geographics* 14: 19. doi: 10.1186/s12942-015-0012-x. PMC4453214 26014352

[R16] Ezran C , Bonds MH, Miller AC et al. 2019. Assessing trends in the content of maternal and child care following a health system strengthening initiative in rural Madagascar : a longitudinal cohort study. *PLoS**Medicine* 16: 1–23. 10.1371/journal.pmed.1002869 PMC6701767 31430286

[R17] Feikin DR , Nguyen LM, Adazu K et al. 2009. The impact of distance of residence from a peripheral health facility on pediatric health utilisation in rural western Kenya. *Tropical Medicine & International Health* 14: 54–61. 19021892 10.1111/j.1365-3156.2008.02193.x

[R18] Fullman N , Barber RM, Abajobir AA et al. 2017. Measuring progress and projecting attainment on the basis of past trends of the health-related Sustainable Development Goals in 188 countries: an analysis from the Global Burden of Disease Study 2016. *Lancet* 390: 1423–59. 28916366 10.1016/S0140-6736(17)32336-X PMC5603800

[R19] Garchitorena A , Miller AC, Cordier LF et al. 2018. Early changes in intervention coverage and mortality rates following the implementation of an integrated health system intervention in Madagascar. *BMJ**Global Health* 3: e000762. 10.1136/bmjgh-2018-000762 PMC6001915 29915670

[R20] Garchitorena A , Miller AC, Cordier LF et al. 2017. In Madagascar, use of health care services increased when fees were removed: lessons for universal health coverage. *Health**Affairs* 36: 1443–51. 10.1377/hlthaff.2016.1419 28784737

[R21] Gething PW , Johnson FA, Frempong-Ainguah F et al. 2012. Geographical access to care at birth in Ghana: a barrier to safe motherhood. *BMC Public Health* 12: 1. 10.1186/1471-2458-12-991 PMC3533981 23158554

[R22] Gething PW , Noor AM, Zurovac D et al. 2004. Empirical modelling of government health service use by children with fevers in Kenya. *Acta**Tropica* 91: 227–37. 10.1016/j.actatropica.2004.05.002 PMC3166847 15246929

[R23] Giedion U , Alfonso EA, Díaz Y. 2013. *The Impact of Universal Coverage Schemes in the Developing World: A Review of the Existing Evidence*. Washington, DC: UNICO Studies Series.

[R24] Government of Madagascar . 2015. *Strategie Nationale sur la**Couverture Sante Universelle, Madagascar*. Antananarivo: Government of Madagascar.

[R25] Hatt L , Johns B, Connor C et al. 2015. *Impact of Health Systems Strengthening on Health*. Bethesda, MD: Health Finance and Governance Project, Abt Associates Inc.

[R26] Hounton S , Chapman G, Menten J et al. 2008. Accessibility and utilisation of delivery care within a Skilled Care Initiative in rural Burkina Faso. *Tropical Medicine & International Health* 13: 44–52. 18578811 10.1111/j.1365-3156.2008.02086.x

[R27] Humanitarian OpenStreetMap Team . 2019. www.hotosm.org, accessed 1 December 2019.

[R28] Ihantamalala FA , Herbreteau V, Revillion C et al. 2020. Improving geographical accessibility modeling for operational use by local health actors. *Int J Health Geogr*. 19: 27. doi: 10.1101/2020.03.09.20033100. PMC7339519 32631348

[R29] Institut National de la Statistique . 2009. *Enquête Démographique et de Santé, Madagascar*. Antananarivo: Institut National de la Statistique.

[R30] Johri M , Ridde V, Heinmüller R, Haddad S. 2014. Estimation of maternal and child mortality one year after user-fee elimination: an impact evaluation and modelling study in Burkina Faso. *Bulletin of the**World Health**Organization* 92: 706–15. 10.2471/BLT.13.130609 PMC4208477 25378724

[R31] Juran S , Broer PN, Klug SJ et al. 2018. Geospatial mapping of access to timely essential surgery in sub-Saharan Africa. *BMJ**Global Health* 3: e000875. 10.1136/bmjgh-2018-000875 PMC6104751 30147944

[R32] Kashima S , Suzuki E, Okayasu T et al. 2012. Association between proximity to a health center and early childhood mortality in madagascar. *PLoS One* 7: e38370. doi: 10.1371/journal.pone.0038370. PMC3366931 22675551

[R33] Kelly C , Hulme C, Farragher T, Clarke G. 2016. Are differences in travel time or distance to healthcare for adults in global north countries associated with an impact on health outcomes? A systematic review. *BMJ Open* 6: 1–9. 10.1136/bmjopen-2016-013059 PMC5178808 27884848

[R34] Kontopantelis E , Doran T, Springate DA, Buchan I, Reeves D. 2015. Regression based quasi-experimental approach when randomisation is not an option: interrupted time series analysis. *BMJ* 350: h2750. 10.1136/bmj.h2750 PMC4460815 26058820

[R35] Kuupiel D , Adu KM, Apiribu F et al. 2019. Geographic accessibility to public health facilities providing tuberculosis testing services at point-of-care in the upper east region, Ghana. *BMC Public Health* 19: 1–12. 31182068 10.1186/s12889-019-7052-2 PMC6558903

[R36] Lagarde M , Palmer N. 2011. The impact of user fees on access to health services in low- and middle-income countries. *Cochrane Database**of Systematic Reviews*. doi: 10.1002/14651858.CD009094. PMC10025428 21491414

[R37] Langlois ÉV , Karp I, De Dieu Serme J, Bicaba A. 2016. Effect of a policy to reduce user fees on the rate of skilled birth attendance across socioeconomic strata in Burkina Faso. *Health Policy**and Planning* 31: 462–71. 10.1093/heapol/czv088 PMC4986241 26453087

[R38] Lankowski AJ , Siedner MJ, Bangsberg DR, Tsai AC. 2014. Impact of geographic and transportation-related barriers on HIV outcomes in sub-saharan Africa: a systematic review. *AIDS**and Behavior* 18: 1199–223. 10.1007/s10461-014-0729-8 PMC4047127 24563115

[R39] Makanga PT , Schuurman N, Von Dadelszen P, Firoz T. 2016. A scoping review of geographic information systems in maternal health. *Int J Gynecol Obstet. International Federation of Gynecology and Obstetrics* 134: 13–7. 10.1016/j.ijgo.2015.11.022 PMC4996913 27126906

[R40] McLaren Z , Ardington C, Leibbrandt M. 2014. Distance decay and persistent health care inequality in South Africa. *BioMed Cent* 14: 541. doi: 10.1186/s12913-014-0541-1. PMC4236491 25367330

[R41] Mills S , Williams JE, Adjuik M, Hodgson A. 2008. Use of health professionals for delivery following the availability of free obstetric care in Northern Ghana. *Maternal and**Child Health**Journal* 12: 509–18. 10.1007/s10995-007-0288-y 17955355

[R42] Munyaneza F , Ntaganira J, Nyirazinyoye L et al. 2018. Community-based accompaniment and the impact of distance for HIV patients newly initiated on antiretroviral therapy: early outcomes and clinic visit adherence in rural Rwanda. *AIDS**and Behavior* 22: 77–85. 10.1007/s10461-016-1658-5 28025738

[R43] Nguyen HT , Zombré D, Ridde V, De Allegri M. 2018. The impact of reducing and eliminating user fees on facility-based delivery: a controlled interrupted time series in Burkina Faso. *Health Policy**and Planning* 33: 948–56. 10.1093/heapol/czy077 30256941

[R44] Noor AM , Amin AA, Gething PW et al. 2006. Modelling distances travelled to government health services in Kenya. *Tropical Medicine and International Health* 11: 188–96. 16451343 10.1111/j.1365-3156.2005.01555.x PMC2912494

[R45] Noor AM , Zurovac D, Hay SI, Ochola SA, Snow RW. 2003. Defining equity in physical access to clinical services using geographical information systems as part of malaria planning and monitoring in Kenya. *Tropical Medicine and International Health* 8: 917–26. 14516303 10.1046/j.1365-3156.2003.01112.x PMC2912492

[R46] Ouma PO , Maina J, Thuranira PN et al. 2018. Access to emergency hospital care provided by the public sector in sub-Saharan Africa in 2015: a geocoded inventory and spatial analysis. *The**Lancet**Global Health* 6: e342–50. 10.1016/S2214-109X(17)30488-6 PMC5809715 29396220

[R47] Pilcher J , Kruske S, Barclay L. 2014. A review of rural and remote health service indexes: are they relevant for the development of an Australian rural birth index? *BMC Health**Services Research* 14: 1–8. 10.1186/s12913-014-0548-7 PMC4265404 25491346

[R48] R Development Core Team . 2011. *R: A Language and Environment for Statistical Computing*. Vienna: R Foundation for Statistical Computing.

[R49] Rosero-Bixby L . 2004. Spatial access to health care in Costa Rica and its equity: a GIS-based study. *Social Science & Medicine* 58: 1271–84. 14759675 10.1016/S0277-9536(03)00322-8

[R50] Ruktanonchai CW , Ruktanonchai NW, Nove A et al. 2016. Equality in maternal and newborn health: modelling geographic disparities in utilisation of care in five East African countries. *PLoS One* 11: 1–17. 10.1371/journal.pone.0162006 PMC4999282 27561009

[R51] Sachs JD . 2012. Achieving universal health coverage in low-income settings. *The Lancet* 380: 944–7. 10.1016/S0140-6736(12)61149-0 22959391

[R52] Stenberg K , Hanssen O, Edejer TTT et al. 2017. Financing transformative health systems towards achievement of the health Sustainable Development Goals: a model for projected resource needs in 67 low-income and middle-income countries. *The**Lancet**Global Health* 5: e875–87. 10.1016/S2214-109X(17)30263-2 PMC5554796 28728918

[R53] Stock R . 2012. Distance and utilization of health facilities in rural Nigeria. *Social Science & Medicine* 17: 563–70. 10.1016/0277-9536(83)90298-8 6879255

[R54] United Nations General Assembly . 2015. *Transforming Our World: The 2030 Agenda for Sustainable Development*, New York, USA: United Nations.

[R55] Weiss DJ , Nelson A, Vargas-Ruiz CA et al. 2020. Global maps of travel time to healthcare facilities. *Nature Medicine* 26: 1835–1838. doi: 10.1038/s41591-020-1059-1. 32989313

[R56] The World Bank . 2012. *Emergency**Support to Critical Education, Health and Nutrition Services Project*, Washington DC: The World Bank.

[R57] The World Bank . World Health Organization and the International Bank for Reconstruction and Development. Tracking Universal Health Coverage: 2017 Global Monitoring Report. 2017. ISBN 978-92-4-151355-5.

[R58] World Bank . 2019. World Bank Open Data. http://data.worldbank.org, accessed 20 December 2019.

[R59] World Health Organization . 2010. *Monitoring the Building Blocks of Health Systems: A Handbook of Indicators and Their Measurement Strategies*, 1–92. Geneva, Switzerland: World Health Organization.

[R60] World Health Organization . 2018a. *Declaration of Astana*. Geneva.

[R61] World Health Organization . 2018b. *WHO Guideline on Health Policy and System Support to Optimize Community Health Worker Programmes*. Geneva: World Health Organization. 30431747

[R62] World Health Organization . 2019. Global Health Observatory Data Repository. http://apps.who.int/gho/data/view.main.95000, accessed 22 December 2019.

[R63] WorldPop . 2017. *Madagascar 100m Population, Version 2*. University of Southampton, Southampton, England.

[R64] Yao J , Agadjanian V. 2018. Bypassing health facilities in rural Mozambique: spatial, institutional, and individual determinants. *BMC Health Services Research* 18: 1–11. 30594198 10.1186/s12913-018-3834-y PMC6311024

[R65] Zombré D , De Allegri M, Ridde V. 2017. Immediate and sustained effects of user fee exemption on healthcare utilization among children under five in Burkina Faso: a controlled interrupted time-series analysis. *Social Science & Medicine* 179: 27–35. 28242542 10.1016/j.socscimed.2017.02.027

