# Supplementary material for: Geographic barriers to achieving universal health coverage: evidence from rural Madagascar
Source: Health Policy Plan. 2021 Jul 31;36(10):1659–70. doi: 10.1093/heapol/czab087 (PMC8597972; doi:10.1093/heapol/czab087)
Supplement: czab087_Supp [file czab087_supp.zip › Supplementary Information_revisions.docx]

**Geographic Barriers to Achieving Universal Health Coverage: evidence from rural Madagascar**

**- Supplementary Information -**

**Table S1.** Summary of HSS Intervention carried out by the MoH-NGO partnership in Ifanadiana District in 2014-2017, based upon TIDieR guidance

| **1. BRIEF NAME** |
| --- |
| Integrated health system strengthening initiative in Ifanadiana district, Madagascar |
| **2. WHY** |
| ***GOAL/RATIONALE:*** To create a model public health district with universal access to care aiming for broad-based population health impact on mortality. Based upon the World Health Organization’s building blocks of Health System Strengthening: 1) service delivery; 2) health workforce; 3) health information systems; 4) medicines and supplies; 5) financing; 6) leadership. |
| **WHAT** |
| **3. *MATERIALS*** (by level of care and above enumerated building blocks)  **At district hospital level**  (1) *Service delivery:* Overall infrastructure upgrades and outfitting for service delivery, bringing inpatient bed capacity from 25 to 40, upgrades to waste management system, specific renovations of the emergency and triage department, pediatric unit, inpatient ward, isolation ward, and laboratory; support to specific service delivery, including emergency care and provision of a network of 3 ambulances, including 2 new fully equipped ones with 24/7 coverage and 12 NGO paramedics for referrals; maternal and obstetrical care; laboratory service were upgraded to include a total of 53 tests, including microscopy and GenExpert for tuberculosis; social support evolved to support all hospitalized and vulnerable patients; launch of intensive care unit for severe acute malnutrition with complications.  (2) *Health workforce:* Staffing of health workers to reach MoH norms through joint MoH-NGO hires of 7 clinicians, including a trauma surgeon and an anesthesiologist, which were integrated into the Ministry of Health staff (long term solution); staffing supplemented further with fulltime presence of 2 NGO doctors and 4 nurses by end of 2017; non-clinical NGO staff including a team of 3 social workers, support staff (janitors, guards, etc.), a laboratory technician, and a radiology technician; ongoing mentorship and frequent trainings of medical staff in key clinical areas, such as emergency medicine and postoperative care.  (3) *Health information systems:* Creation of a hospital-based M&E team to follow progress of activities and improve quality of HMIS data; implementation of a system for baseline and follow-up facility readiness surveys.  (4) *Medicines and supplies:* Supply chain management and reduction of stock-outs, initially through frequent donations which evolved into a reimbursement program paired with pharmacy management training; provision of medical and non-medical equipment for service delivery, including full laboratory capacity. The NGO became the procurement manager for the hospital pharmacy as of October 2017.  (5) *Financing:* Cost of outpatient and inpatient care fully covered for patients referred by district-wide health centers and self-referred patients who necessitated urgent inpatient care (over 76,000 patients between 2014 and end of 2017); cost of referral to and care at higher levels of care (e.g. university hospital) fully covered for services not available at district hospital.  (6) *Leadership:* Creation of a joint MoH-NGO executive committee for hospital management and transparency; creation of sub committees for specific projects such as infection control or quality of care.  **At health center level**  (1) *Service delivery:* Overall infrastructure renovations and/or extensions for service delivery at 5 target health centers, including ensuring electricity, water, waste management/sterilization capacity, proper pharmacy conditions; provision of medical and non-medical equipment, including beds, armoires, furniture; support to launching specific service delivery of Integrated Management of Childhood Illnesses and malnutrition protocols for every child under 5 attending the health center; ensuring timely referrals and emergency care. Launch of supervision efforts and quality of care improvement projects with a focus on IMCI and malnutrition.  (2) *Health workforce:* Staffing through joint MoH-NGO hires to bring all 13 primary care health centers up to MoH norms (1 doctor, 1 nurse, 1 midwife, 1 dispenser, 1 support staff at each facility); 33 MOH-NGO hires were integrated into the Ministry of Health staff (long term solution); at target health facilities, hiring exceeded norms and NGO clinicians were permanently present (~2 clinicians per health center) to implement service delivery protocols (e.g. IMCI, malnutrition); trainings for medical staff (some district-wide) such as obstetrical and neonatal care; ongoing supervision and mentorship in target centers for IMCI and malnutrition.  (3) *Health information systems:* Joint MoH-NGO training and supervision to improve HMIS data quality (district-wide); implementation of system for baseline and follow-up of facility readiness surveys.  (4) *Medicines and supplies:* Supply chain management and reduction of stock-outs, initially through frequent donations which evolved into a reimbursement program paired with pharmacy management training.  (5) *Financing:* Essential medicines and consumables provided free of charge to all patients (more than 130,000 patients between October 2014 launch and end of 2017); full details of this program are available at Garchitorena et al. (2017).  (6) *Leadership:* Close collaboration with district health managers for the planning and implementation of activities.  **At community level**  (1) *Service delivery:* Construction of 21 community health posts; specific service delivery in Integrated Management of Childhood Illnesses and malnutrition protocols for every child under 5, community sensitization and mass testing, urgent care, and mobile clinics with direct care provision by NGO clinicians every other month. In 2017 the NGO started to support monthly supervision of CHWs at the health facilities.  (2) *Health workforce:* 14 active CHW supervisors – NGO staff that are training, coaching and monthly supervision of ~86 community health workers by mobile teams of trained nurses by the end of 2017; community IMCI training provided for CHWs in all of the intervention area  (3) *Health information systems:* Joint MoH-NGO training to improve HMIS data quality on community health.  (4) *Medicines and supplies:* Monthly provision and follow-up of MNCH medicine stocks, including malaria diagnosis and treatment, oral rehydration salts, NSAIDS, antibiotics and zinc.  (5) *Financing:* cost of MNCH medicine stocks fully covered; financial and non-financial incentives to CHWs and local leadership.  (6) *Leadership:* Community engagement and participation (e.g. community health posts are built by the community, with NGO support for roofing, painting, furniture and equipment).  **4. *PROCEDURES***  All interventions were aimed at fulfilling existing Madagascar Ministry of Health protocols and standards. |
| **5. WHO PROVIDED** |
| **At district hospital level**  Ministry of Health clinicians provided the majority of service delivery. NGO clinicians are integrated in the hospital staff and provide direct care as any other clinician during external consultations and clinical rounds, but also carry out frequent training. Non-clinical NGO staff provided social support to vulnerable patients, helped manage the patient circuit to benefit from fee exemptions (registration, validation).  **At health center level**  Ministry of Health doctors and nurses provided the majority of service delivery. By MoH norms each health center (CSB2) should have 1 doctor, 1 nurse, 1 midwife, 1 dispenser, and 1 support staff. Additional NGO clinicians (~2 per health facility) provided some direct care, especially for the implementation of malnutrition and IMCI protocols, but focused mostly on training and supervision.  **At community level**  Two community health workers per fokontany (a cluster of villages, lowest administrative unit) provided basic MNCH care, supervised monthly by the clinicians of their respective health center. NGO mobile teams of nurses provided on-site mentoring and supervision of CHWs every two months. They also provided direct care at community level during on-site supervisions for fokontany located >10 kilometers from a health center. |
| **6. HOW (modes of delivery)** |
| NGO employees worked in partnership with existing networks of MoH clinicians and community health workers within existing public health facilities. Wherever possible, such as in the case of supply chain management, leadership and financing, the intervention deliberately avoids the creation of parallel systems of care. |
| **7. WHERE** |
| **At district hospital level**  The initial HSS catchment area comprised the only district hospital, located in Ifanadiana city. Most referrals to higher levels of care (tertiary) were sent to the university hospital in Fianarantsoa (2h away by car), and some to specialized facilities in Antananarivo (capital, ~1 day by car).  **At health center level**  Full health center activities were implemented in the health centers of the five communes closest to the hospital on the district’s sole paved road (i.e. Ranomafana, Kelilalina, Ifanadiana, Tsaratanana, Antaretra); all 13 health centers in the district received trainings, staffing support to reach MoH norms, and some access to the referral network (limited by accessibility).  **At community level**  By the end of 2017, community activities had been rolled out in fokontany from four of the five communes within the HSS catchment area (43 out of a total of 195 total fokontany in the district). |
| **8. WHEN AND HOW MUCH** |
| All interventions were progressively rolled out during the study period.  The earliest intervention activities implemented (starting in April-May 2014, at the beginning of the study period) included the ambulance network, staffing of health centers and district hospital, and provision of medical equipment in four communes.  The renovation of health centers also began in April-May 2014 but the date of completion varied for each health facility.  The renovation of the emergency and triage unit and pediatric guard at the district hospital were completed by early 2016.  Removal of user fees at health centers and hospital began in October of 2014.  Implementation of IMCI and malnutrition protocols at all health centers began in October 2015.  Community-level activities began in November 2015 in two communes, with an expansion to four communes in February 2017.  First expansion of the HSS intervention area at health facility level to include a fifth commune in October 2017. |
| **9. TAILORING** |
| N/A |
| **10. MODIFICATIONS** |
| N/A; The intervention is progressively being implemented, as explained in section 8 (when and how much) |
| **HOW WELL** |
| N/A; The aim of this study was to study the evolution of geographic access to primary care in Ifanadiana District. Full details of the impact assessment are available in the main text. |

**Table S2.** Univariate model results (Generalized linear mixed models with random intercept at the PHC closest to the Fokontany of residence)

| **Variable** | **Outpatient visits for all ages** | | **Outpatient visits for children under age 5** | | **Outpatient visits for all ages, excluding malaria** | | **Outpatient visits for children under age 5, excluding malaria** | |
| --- | --- | --- | --- | --- | --- | --- | --- | --- |
|  | OR (95% CI) | AIC | OR (95% CI) | AIC | OR (95% CI) | AIC | OR (95% CI) | AIC |
| **Geographic factors** |  |  |  |  |  |  |  |  |
| Network distance to PHC (every 10km) | 0.34 (0.338-0.342) | 161214 | 0.379 (0.375-0.383) | 73705 | 0.329 (0.327-0.332) | 124945 | 0.345 (0.34-0.35) | 56886 |
| Network distance to PHC (every 10km, linear term in bivariate model) | 0.034 (0.033-0.035) | 155714 | 0.067 (0.063-0.072) | 73025 | 0.026 (0.025-0.027) | 119380 | 0.046 (0.043-0.05) | 56162 |
| Network distance to PHC (every 10km, quadratic term in bivariate model) | 2.343 (2.295-2.392) | - | 1.675 (1.615-1.739) | - | 2.701 (2.637-2.766) | - | 1.94 (1.854-2.03) | - |
| Time to PHC (every 2 hours) | 0.4 (0.398-0.402) | 167761 | 0.441 (0.436-0.445) | 75489 | 0.389 (0.387-0.392) | 129802 | 0.405 (0.4-0.41) | 58090 |
| Time to PHC (every 2 hours, linear term in bivariate model) | 0.057 (0.055-0.058) | 162140 | 0.105 (0.1-0.111) | 74860 | 0.046 (0.044-0.047) | 124180 | 0.076 (0.072-0.081) | 57426 |
| Time to PHC (every 2 hours, quadratic term in bivariate model) | 1.851 (1.824-1.878) | - | 1.43 (1.393-1.468) | - | 2.032 (1.999-2.066) | - | 1.582 (1.532-1.634) | - |
| **Health system factors** |  |  |  |  |  |  |  |  |
| Number of health staff | 1.144 (1.14-1.148) | 292584 | 1.076 (1.069-1.083) | 111971 | 1.222 (1.217-1.227) | 219482 | 1.159 (1.15-1.168) | 86011 |
| Major PHC (vs basic PHC) | 3.395 (1.465-7.869) | 298080 | 3.65 (1.756-7.587) | 112465 | 3.351 (1.356-8.28) | 228574 | 3.8 (1.733-8.332) | 87445 |
| HSS catchment (vs. outside) | 0.996 (0.974-1.018) | 298087 | 0.838 (0.808-0.87) | 112386 | 1.054 (1.027-1.081) | 228564 | 0.815 (0.781-0.852) | 87370 |
| **Impact of HSS programs** |  |  |  |  |  |  |  |  |
| User fee exemption program | 1.715 (1.693-1.737) | 290919 | 1.285 (1.257-1.313) | 111955 | 2.072 (2.04-2.104) | 219271 | 1.439 (1.401-1.477) | 86689 |
| Community health program | 1.615 (1.597-1.632) | 290576 | 1.263 (1.237-1.289) | 111973 | 1.917 (1.893-1.941) | 218245 | 1.489 (1.454-1.525) | 86386 |
| **Underlying trends** |  |  |  |  |  |  |  |  |
| Linear trend (year) | 1.086 (1.082-1.09) | 295892 | 1.003 (0.997-1.009) | 112473 | 1.192 (1.187-1.196) | 221372 | 1.1 (1.092-1.108) | 86779 |
| Seasonal trend | 1.323 (1.316-1.331) | 288281 | 1.337 (1.324-1.35) | 108989 | 1.022 (1.016-1.029) | 228535 | 1.152 (1.14-1.166) | 86843 |
| Lagged trend (1-month lag) | 1.935 (1.929-1.942) | 154511 | 1.542 (1.535-1.549) | 69557 | 2.39 (2.377-2.402) | 121957 | 1.748 (1.736-1.759) | 58052 |

**
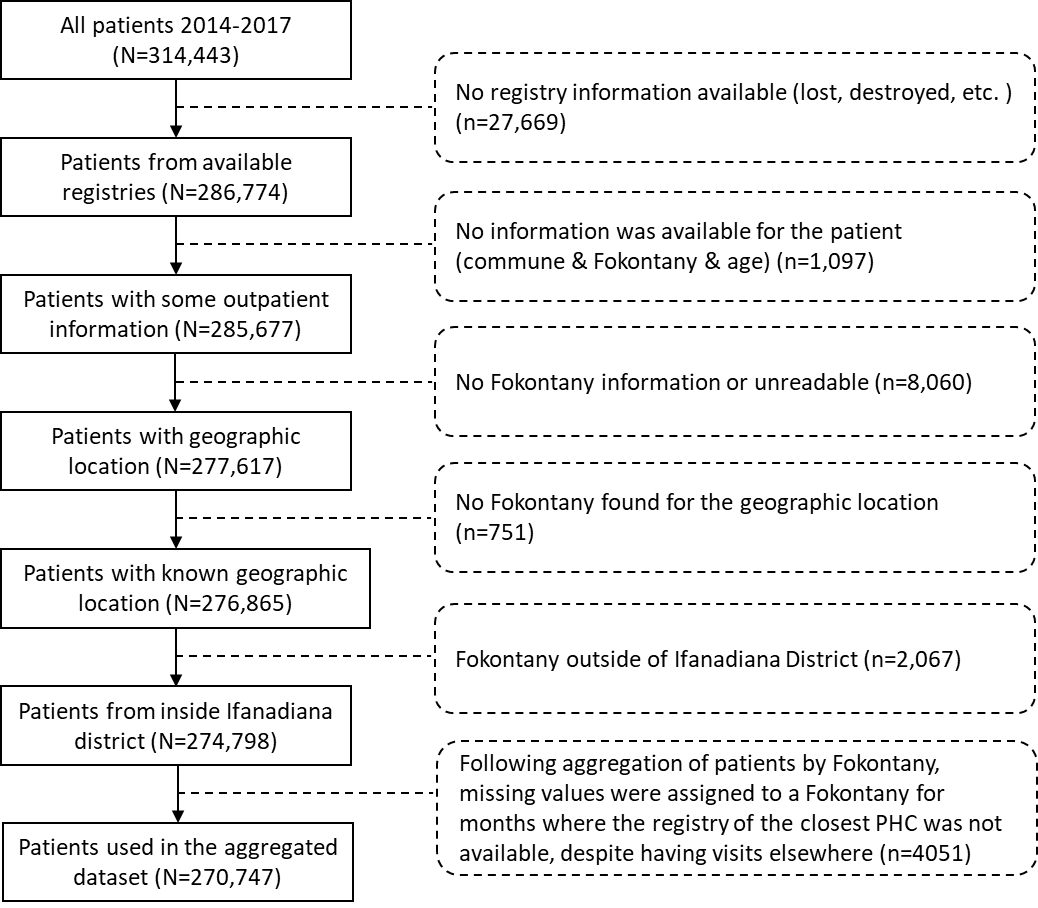
**

**Figure S1. Patient data available for geographic analyses from the 19 PHC in Ifanadiana District, 2014-2017.** Diagram shows the information loss from the total number of patients that were reported by PHC to the Ministry of Health for the study period, to the final database used in the geographic analyses. The final database contained 86.1% of all patients reported. We excluded from the analysis a new health center inaugurated in November 2016 because it barely had any attendance during our study period (one third of the PHC district average), so patients were most likely attending their previous reference PHC.


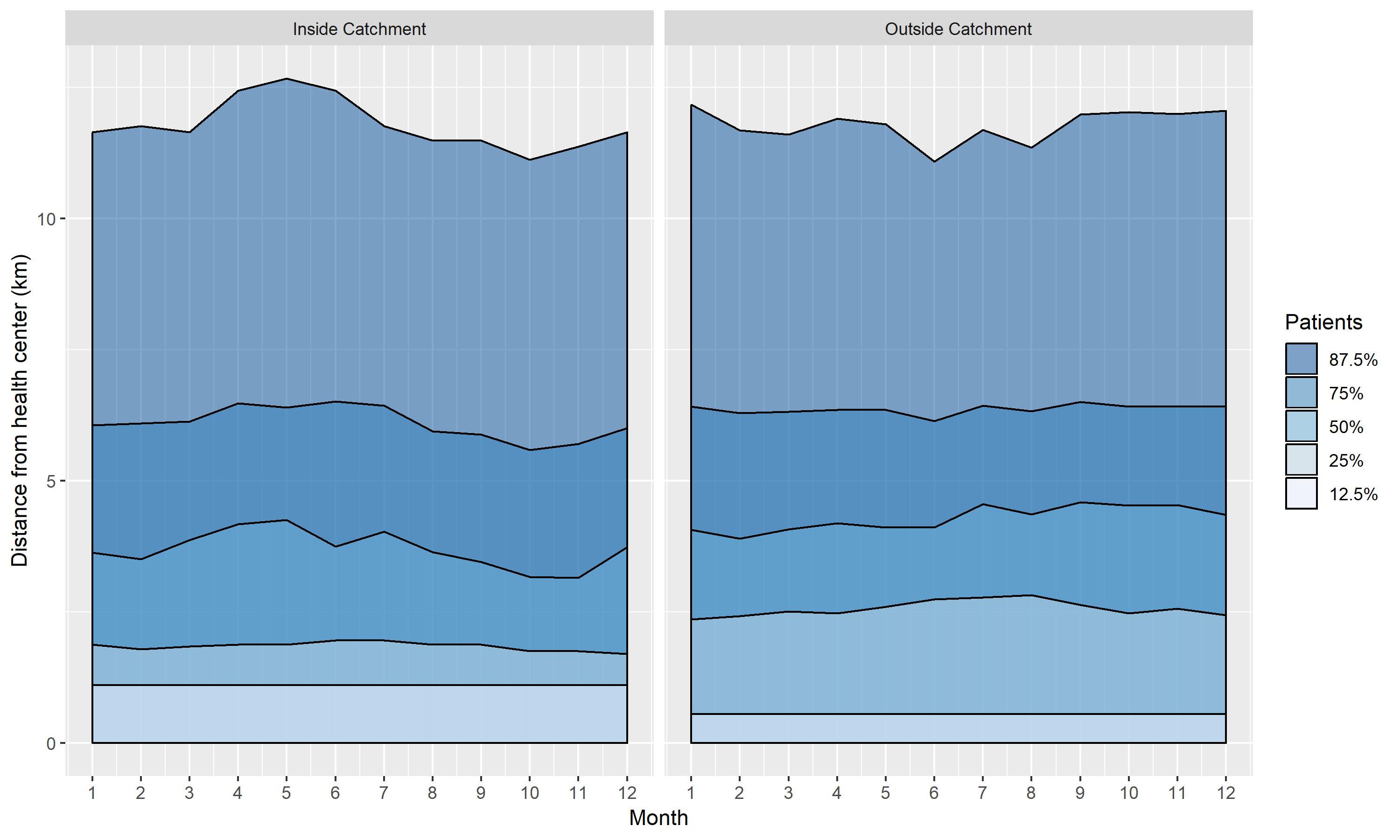


**Figure S2. Distance travelled by patients in Ifanadiana District at each month of the year, 2014-2017.** Color shades represent the percentiles of distance travelled by patients who attended a PHC in the HSS intervention catchment (left) and the rest of Ifanadiana District (right). The graph reveals that in the HSS intervention catchment, 50% of patients come from within 2.2 km and 75% come from within 4km, with seasonal patterns that are most notable at further distances. No seasonal patterns are observed outside of the intervention catchment, where utilization rates are significantly lower.

**
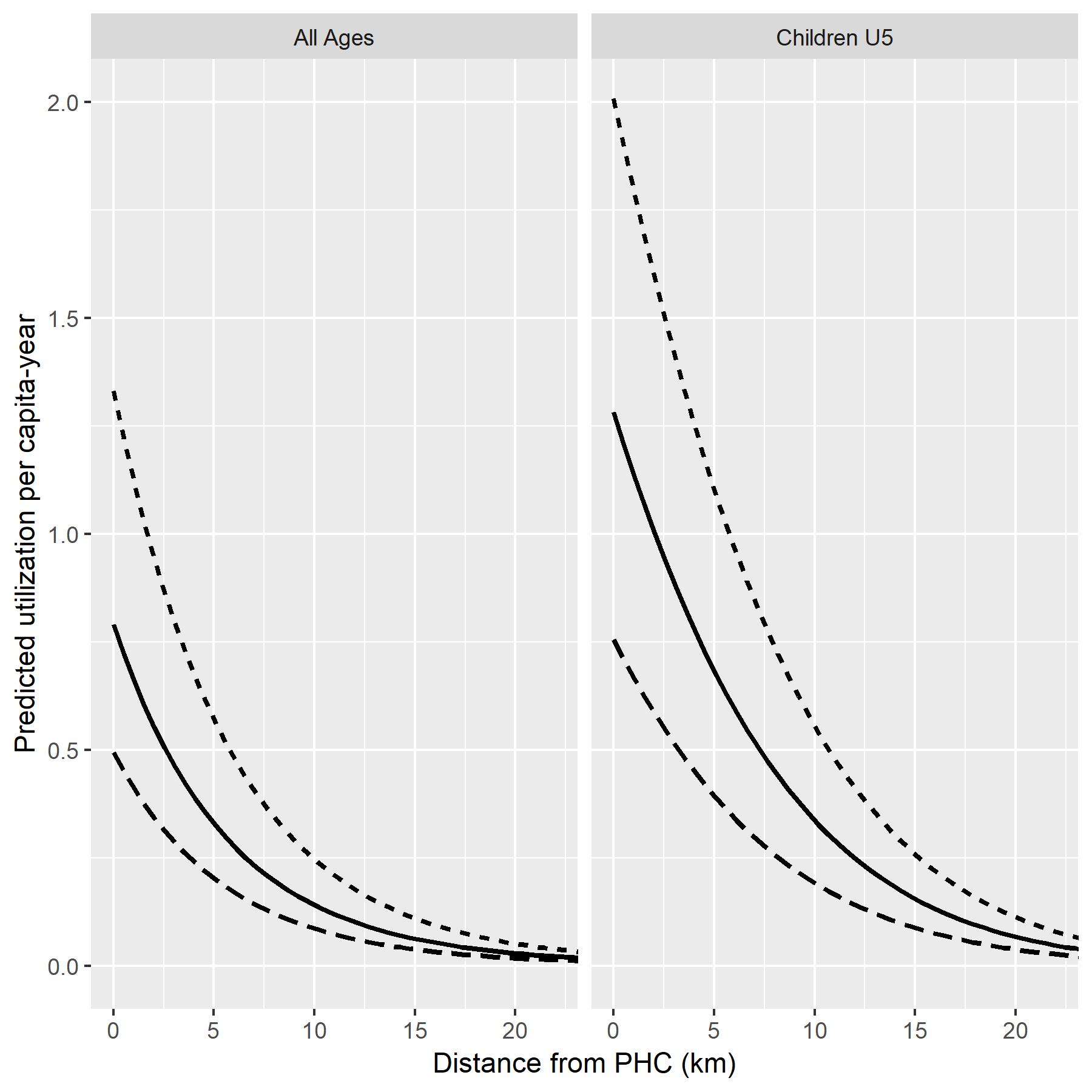
**

**Figure S3. Predicted non-linear relationship between PHC utilization and distance from PHC in multivariate models.** Relationships shown here correspond to results outlined in Table 3 in the manuscript. Solid lines represent the average relationship between utilization and distance from PHC, excluding random effects and holding all variables at their average values. Dashed lines represent the same relationship between utilization and distance from PHC, but including random effects for a PHC with higher and lower utilization than average.

**
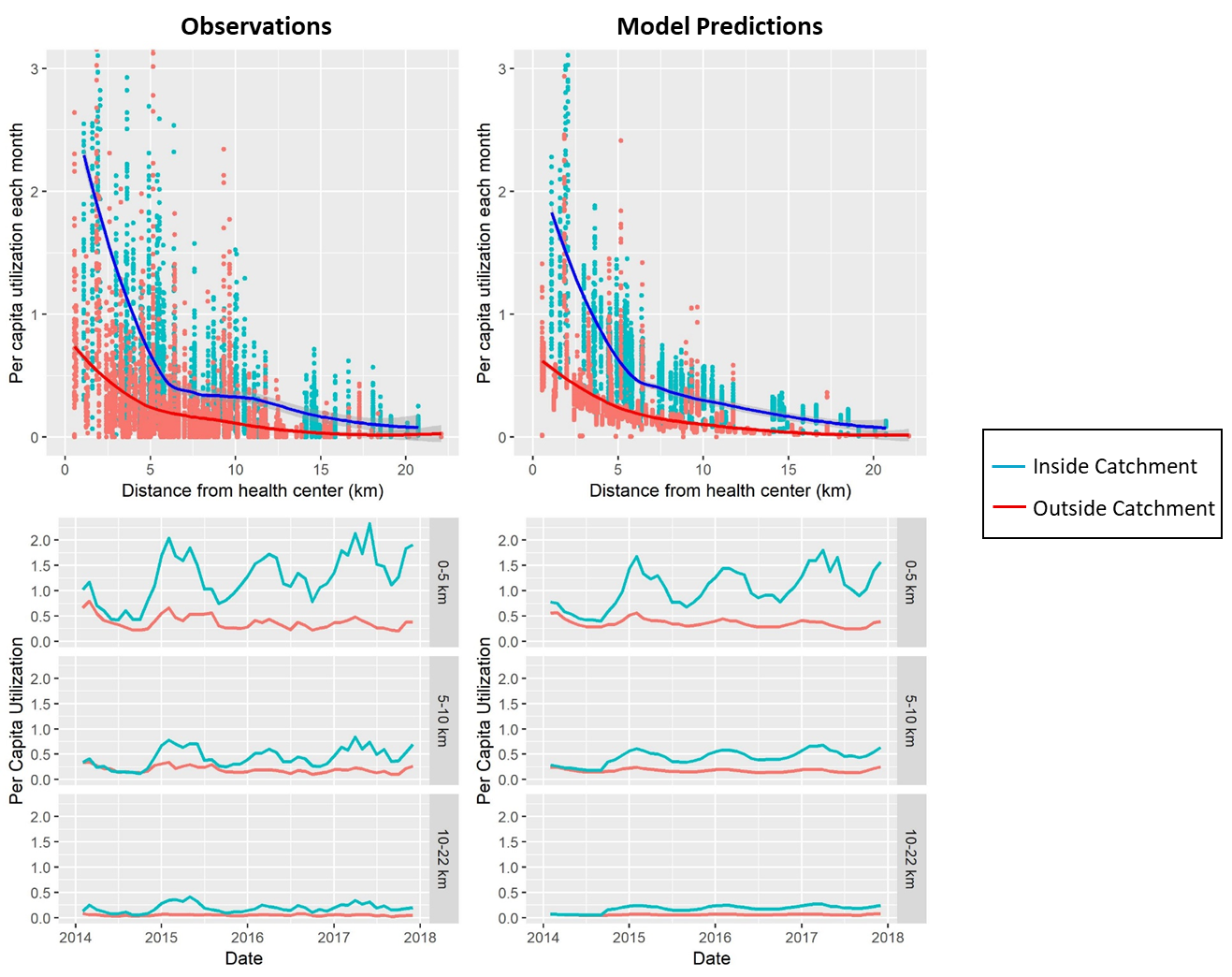
**

**Figure S4. Comparison of observed utilization patterns and multivariate model predictions.** Results show that our model predicted well geographical and temporal utilization patterns at different distances, both inside and outside the intervention catchment, during the study period. Results were annualized to improve comparability, and utilization values above 3 in graphs at the top were removed to improve visualization.

**
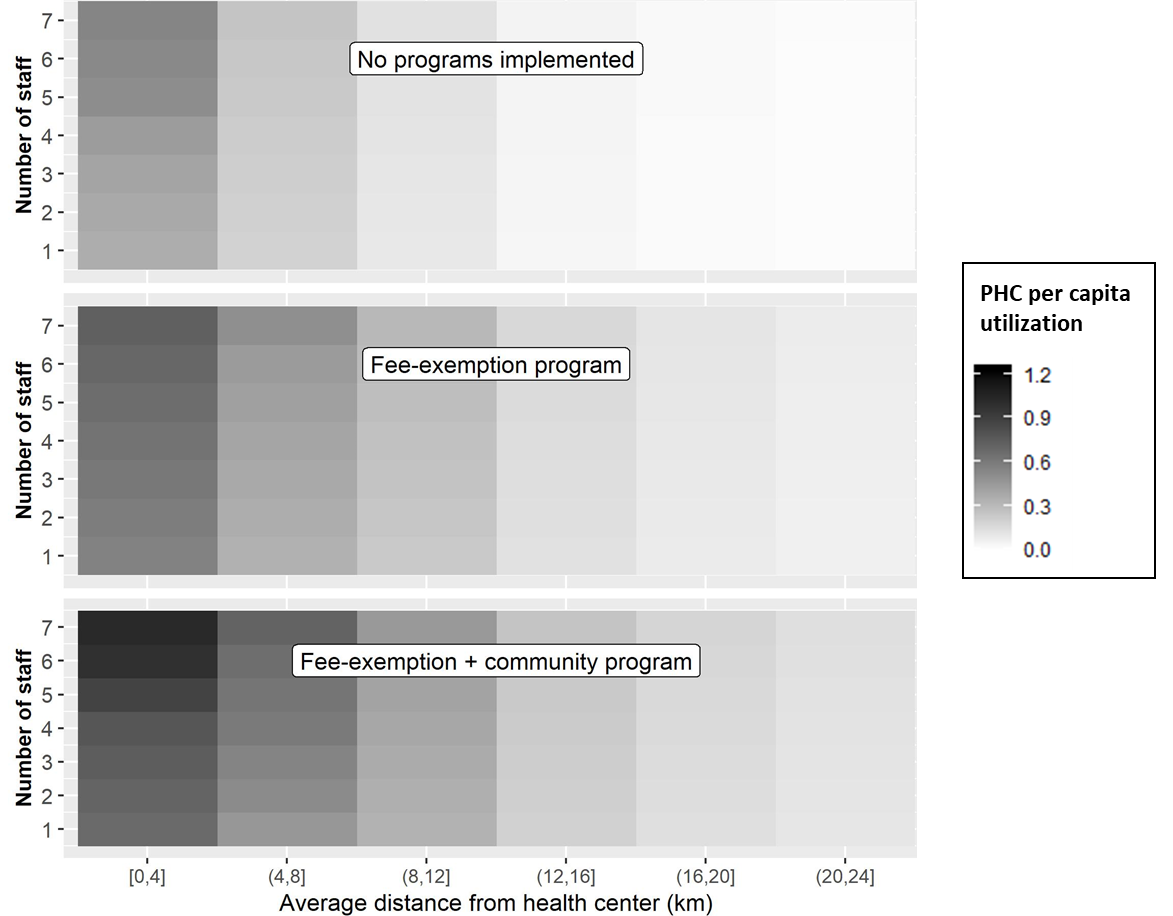
**

**Figure S5. Model predictions of yearly PHC per capita utilization in Ifanadiana district under different HSS scenarios.** Results show that, in scenarios where additional staff is hired, point-of-care fees are removed, and community health workers are supported to diagnose and refer patients, PHC utilization increases substantially. However, the effect of distance to a PHC remains the major limitation and these effects are largely diluted after the first few kilometres. We did not find a HSS scenario where populations living further than 4km had over 1 visit per capita-year.


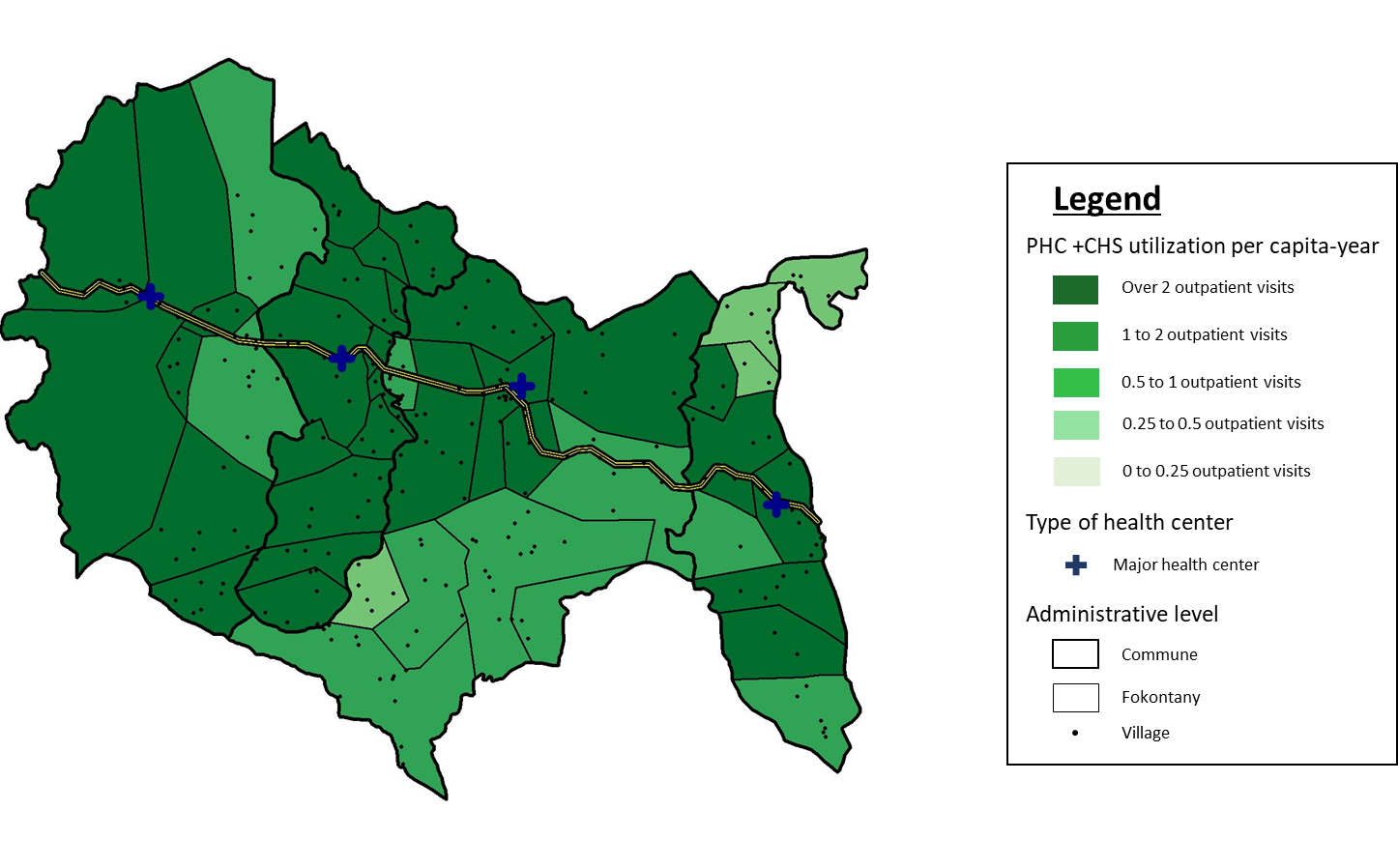
**Figure S6. Geographical distribution of combined PHC and CHS per capita utilization for children under 5 years in the HSS catchment.** Color shades represent annual per capita visits for the year 2017 in the four communes with community health support by the NGO. Map reveals a more homogeneous pattern according to distance to PHC, and high levels of utilisation (over 1 visit per capita-year) for the majority of populations.
